# Immunoglobulin superfamily member 3 is required for the vagal neural crest cell migration and enteric neuronal network organization

**DOI:** 10.1038/s41598-023-44093-8

**Published:** 2023-10-11

**Authors:** Jayendrakishore Tanjore Ramanathan, Tomáš Zárybnický, Pauliina Filppu, Hector J Monzo, Outi Monni, Topi A Tervonen, Juha Klefström, Laura Kerosuo, Satu Kuure, Pirjo Laakkonen

**Affiliations:** 1 Translational Cancer Medicine Research Program, Faculty of Medicine, University of Helsinki, Helsinki, Finland; 2 Stem Cells and Metabolism Research Program, Faculty of Medicine, University of Helsinki, Helsinki, Finland; 3 Applied Tumor Genomics Research Program, Faculty of Medicine, University of Helsinki, Helsinki, Finland; 4 Finnish genome editing center (FinGEEC), Helsinki Institute of Life Science (HiLIFE), University of Helsinki, Helsinki, Finland; 5 Finnish Cancer Institute & FICAN South, Helsinki University Hospital (HUS), Helsinki, Finland; 6 Neural Crest Development and Disease Unit, Department of Health and Human Services, National Institute of Dental and Craniofacial Research, National Institutes of Health, Bethesda, MD 20892 USA; 7 GM-unit, Helsinki Institute of Life Science (HiLIFE), University of Helsinki, Helsinki, Finland; 8 iCAN Flagship Program, University of Helsinki, Helsinki, Finland; 9 Laboratory Animal Centre, Helsinki Institute of Life Science (HiLIFE), University of Helsinki, Helsinki, Finland

**Keywords:** Embryogenesis, Cell adhesion, Cell migration, Development of the nervous system, Peripheral nervous system

## Abstract

The immunoglobulin (Ig) superfamily members are involved in cell adhesion and migration, complex multistep processes that play critical roles in embryogenesis, wound healing, tissue formation, and many other processes, but their specific functions during embryonic development remain unclear. Here, we have studied the function of the immunoglobulin superfamily member 3 (IGSF3) by generating an *Igsf3* knockout (KO) mouse model with CRISPR/Cas9-mediated genome engineering. By combining RNA and protein detection methodology, we show that during development, IGSF3 localizes to the neural crest and a subset of its derivatives, suggesting a role in normal embryonic and early postnatal development. Indeed, inactivation of *Igsf3* impairs the ability of the vagal neural crest cells to migrate and normally innervate the intestine. The small intestine of *Igsf3* KO mice shows reduced thickness of the muscularis externa and diminished number of enteric neurons. Also, misalignment of neurons and smooth muscle cells in the developing intestinal villi is detected. Taken together, our results suggest that IGSF3 functions contribute to the formation of the enteric nervous system. Given the essential role of the enteric nervous system in maintaining normal gastrointestinal function, our study adds to the pool of information required for further understanding the mechanisms of gut innervation and etiology behind bowel motility disorders.

## Introduction

Neural crest (NC) cells are a transient, highly migratory population of stem cells that forms during early embryogenesis in between the neural plate and the non-neural ectoderm after gastrulation^1–3^. After specification in the dorsal neural tube, NC cells undergo an epithelial-to-mesenchymal transition (EMT), delaminate from the neural epithelium, and migrate to various parts of the embryo where they differentiate to a diverse set of cell types. These include neurons and glia of the sensory, autonomic and enteric nervous systems, endocrine cells like the epinephrine producing chromaffin cells of the adrenal gland, epidermal pigment forming melanocytes, and various skeletal and connective tissue components of the facial skeleton and the neck region^2–4^.

The migration of NC cells to their respective destinations depends on environmental cues such as chemoattraction and repulsion, as well as mechanical cues, so the interactions between NC cells and non-neural ectoderm and surrounding mesenchyme are heavily reliant on both cell–cell and cell–matrix interactions^5, 6^. The regulation of the adhesion properties is strict as both too weak and too strong adhesion leads to migration defects^7^. Thus, cell adhesion molecules play a vital role in NC cell migration and aggregation at their destinations, which is a concerted action regulated by the immunoglobulin superfamily (IgSF) members of cell adhesion molecules (IgCAM), cadherins, integrins, and the proteolytic enzymes^8^. Importantly, the involvement of the EWI subfamily of IgSF molecules (containing the conserved ectodomain Glu-Trp-Ile (EWI) motifs) in NC cell biology is currently unknown.

The IgSFs form a large family of cell surface proteins that are involved, in addition to the signaling and immune associated functions, in adhesion-mediated cellular processes such as recognition, formation of cell-to-cell and cell-to-ECM binding complexes and motility^9, 10^. In our study, we have focused on the function of the IgSF member 3 (IGSF3) by generating and analyzing a CRISPR/Cas9 knockout (KO) mouse model. The gene encoding *Igsf3*, an EWI-subfamily member of IgSF, is located in chromosome one, and based on its sequence, it is a putative membrane protein with predicted functions in cell adhesion and cell surface receptor signaling with no predicted role in immune system regulation^11^. However, the physiological role of IGSF3 is largely unknown. Recently, *Igsf3* was reported to be involved in axonal growth and branching of granule cells in the developing cerebellum^12^.

The lining of the digestive tract is exposed to a broad range of chemicals and organisms, and the gut is comprised of numerous sensors that in addition to sensing nutrients, also serves as a first-line defense to detect micro-organisms and toxins. As part of the peripheral nervous system, the enteric nervous system (ENS) plays a pivotal part in communicating the sensory information into extensive endocrine, neural, immune, and non-immune responses^13^. Due to the direct connection between the ENS and the central nervous system (CNS) and the ability of these neuronal connections to serve as gateways for disease transmission, dysfunctions of the ENS are not only associated with digestive disorders but also neurological disorders^14^. The ENS development is necessary to warrant the regulation of gut function^15^. Four general concentric layers make up the gastrointestinal tract: from the innermost lumen facing mucosa, followed by submucosa, muscularis externa (muscularis propria), and the outermost protective serosa. Additionally, each layer consists of structural and functional subcomponents^16–19^. The epithelium, lamina propria, and muscularis mucosae make up the mucosa; the submucosal plexus constitutes the submucosa. Furthermore, there are three segments of the muscularis externa: a circular smooth muscle layer, a myenteric plexus, and a longitudinal smooth muscle layer^18–20^. Thus, two plexuses compose the ENS: the myenteric plexus sits between the outer longitudinal smooth muscle cell layer and the inner circular smooth muscle layer; the submucosal plexus sits between the inner circular smooth muscle cell layer and the epithelial/mucosal layers of the gut^16, 17, 21, 22^.

Here, we demonstrate that during development IGSF3 is expressed in the neural crest and its derivatives, the enteric nervous system neurons. By generating and analyzing the *Igsf3* KO mice we show that *Igsf3* is required for a faithful NC cell migration and proper development of the small intestine.

## Results

### IGSF3 is expressed in the developing mouse nervous system and neural crest derivatives

**Figure 1:**
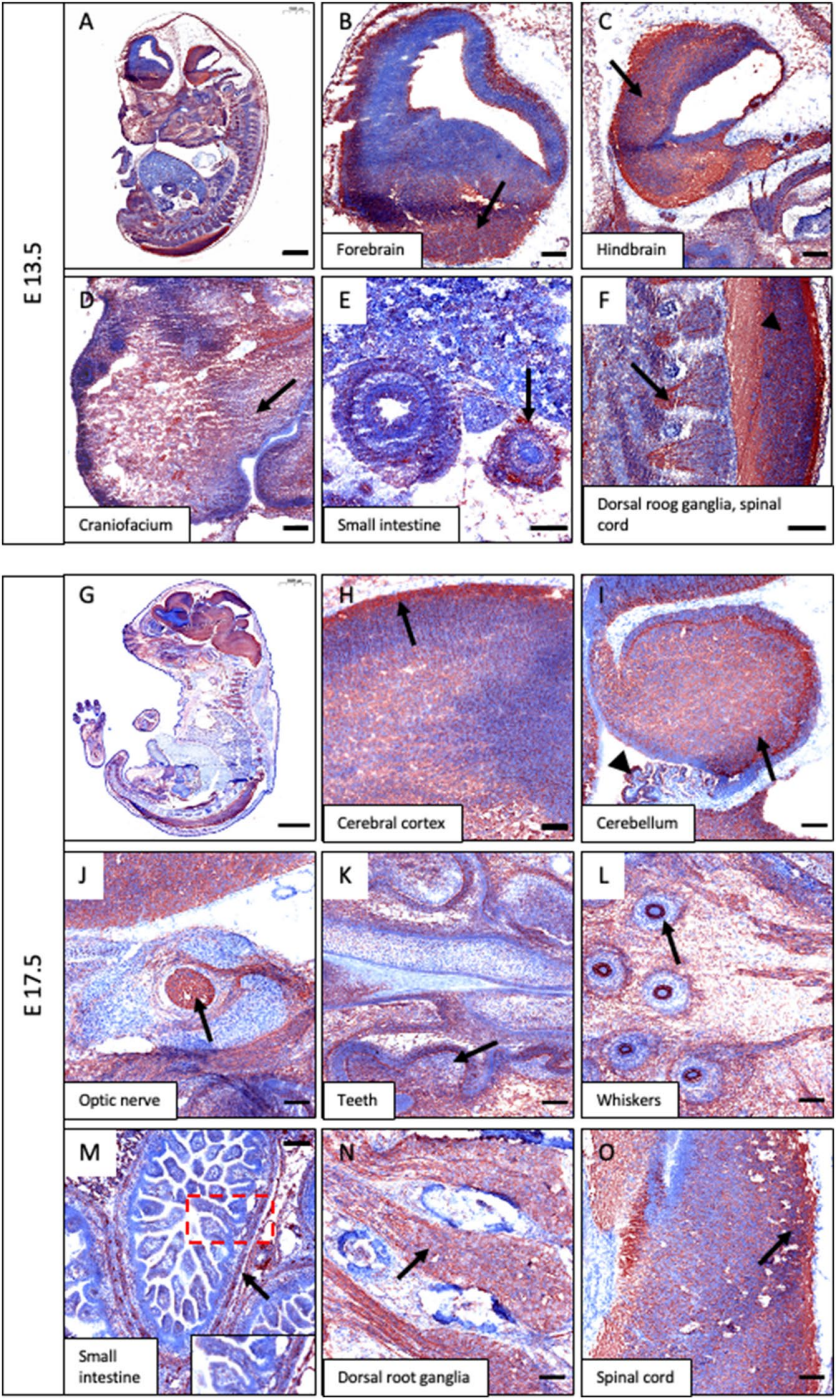
IGSF3 expression in the developing mouse organs. (**A**–**F**) Sagittal sections of mouse embryo at E13.5 stained with an anti-IGSF3 antibody and visualized in brown color. (**A**) Overall IGSF3 protein expression in the embryo. (**B**–**F**) Higher magnification panels show expression (marked by arrows) in the forebrain (**B**), the hindbrain (**C**), the craniofacium (**D**), small intestine (**E**), and dorsal root ganglia (arrow) and spinal cord (arrowhead) (**F**). (**G**–**O**) Sagittal sections of mouse embryo at E17.5 stained with anti-IGSF3 antibody and visualized in brown color. (**G**) Overall expression in the embryo. (**H**–**O**) Higher magnification panels show expression (marked by arrows) in the cerebral cortex of the brain (**H**), the cerebellum (arrow) and the choroid plexus (arrow head) (**I**), optic nerve (**J**), teeth (**K**), whiskers (arrow) and the craniofacium (**L**), developing enteric ganglionic plexi of the small intestine, insert shows higher magnification of the boxed area (**M**), dorsal root ganglia (**N**), and the spinal cord (**O**). Scale bars: (**A**) 1000 µm; (**B**–**F**) 200 µm; (**G**) 2000 µm; (**H**–**O**) 100 µm.

To examine the IGSF3 expression during fetal development, mouse embryos (E13.5 and E17.5) were immunostained with an anti-IGSF3 antibody (Fig. 1A,G). The highest levels of IGSF3 protein expression were detected in the NC-derived tissues and parts of the CNS, in agreement with the previously reported mRNA expression pattern in the brain^12^. Specifically, IGSF3 protein expression was found in the brain cortex (Fig. 1B,C,H), cerebellum (Fig. 1I), and optic nerve (Fig. 1J), and in multiple NC-derived tissues including teeth (Fig. 1K), the craniofacial skeleton (Fig. 1D,L), and developing enteric ganglionic plexi of the small intestine and in the intestinal villi (Fig. 1E,M, also note the higher magnification insert in M). Similarly, the dorsal root ganglia (Fig. 1F,N) and the spinal cord (Fig. 1F,O) expressed IGSF3.

### Single-cell RNAseq analysis identifies *Igsf3* expression in the neural crest

**Figure 2:**
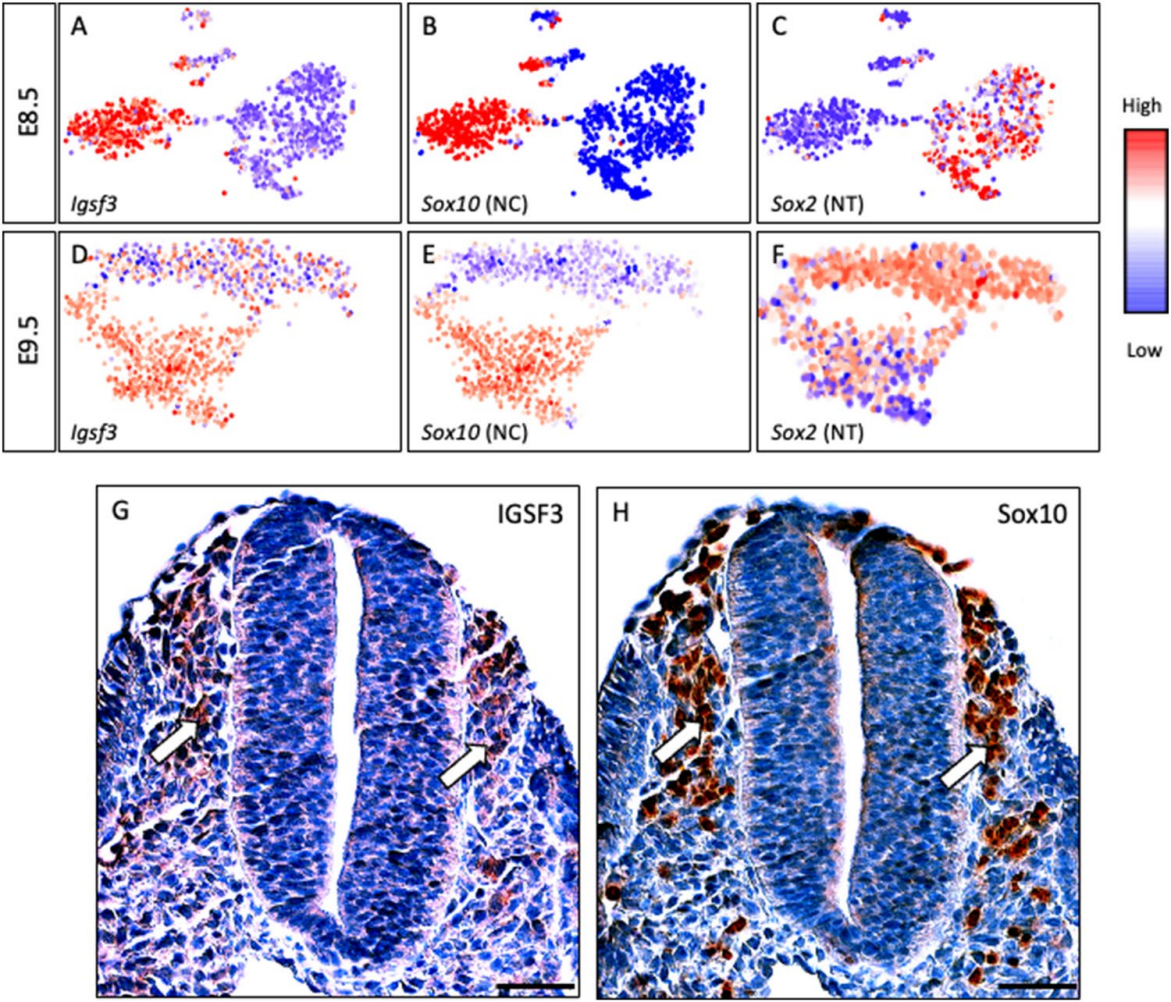
Both IGSF3 mRNA and protein are expressed in the neural crest cells. (**A**–**F**) Single cell RNA sequencing analysis at E8.5 and E9.5 represented as t-SNE plots reveals the neural crest (NC) and neural tube (NT) cell clusters defined by *Sox10* (**B**,**E**) and *Sox2* (**C**,**F**) expression, respectively. The subclusters are assigned according to the annotations from the original analysis of the source data^23^. *Igsf3* is expression is restricted to the neural crest cells at E8.5 (**A**). At E9.5, *Igsf3* is expressed predominantly by the delaminating and migrating neural crest which is identified by *Sox10* with some expression in the neural tube cells (**F**). (**G**,**H**) Serial sections of E9.5 embryo were stained using the anti-IGSF3 and anti-SOX10 antibodies (red color). IGSF3 expression (**G**) is detected in the Sox10-positive (**H**) neural crest cells as indicated by arrows pointing to the neural crest cells that are newly migrating out of the dorsal neural tube on both sides. Scale bars: (**G**,**H**) 50 µm.

The high expression of IGSF3 in the NC lineage intrigued us to explore the published single-cell RNA (scRNA) sequencing datasets of mouse NC (E8.5 and E9,5; *Wnt1-Cre;* R26^TOM^background)^23^. At E8.5, cells separate into NC (identified by *Sox10*) and the CNS forming neural tube (NT, identified by the high expression of the neural progenitor marker *Sox2*) clusters. The NC cell cluster showed high expression levels of *Igsf3* (Fig. 2A–C). Furthermore, the expression pattern continued at E9.5*,* with the highest *Igsf3* expression mostly detected in the *Sox10* + NC cells with some expression also in the cells positive for *Sox2* (Fig. 2D–F). Part of the *Sox2-*positive cells may represent NC cells as lower *Sox2* expression is typical for the premigratory NC cells^24^. At this point, the cranial NC in the *Sox10*-positive population has already reached the late migratory stage, whereas the vagal and anterior trunk NC is still emigrating or early migratory^23^. To verify these findings at the protein level, we stained the E9.5 embryos with anti-IGSF3 and anti-Sox10 antibodies. Indeed, high IGSF3 expression was detected in the Sox10-positive premigratory and migratory neural crest region when analyzed in subsequent sections (Fig. 2G and H).

**Figure 3:**
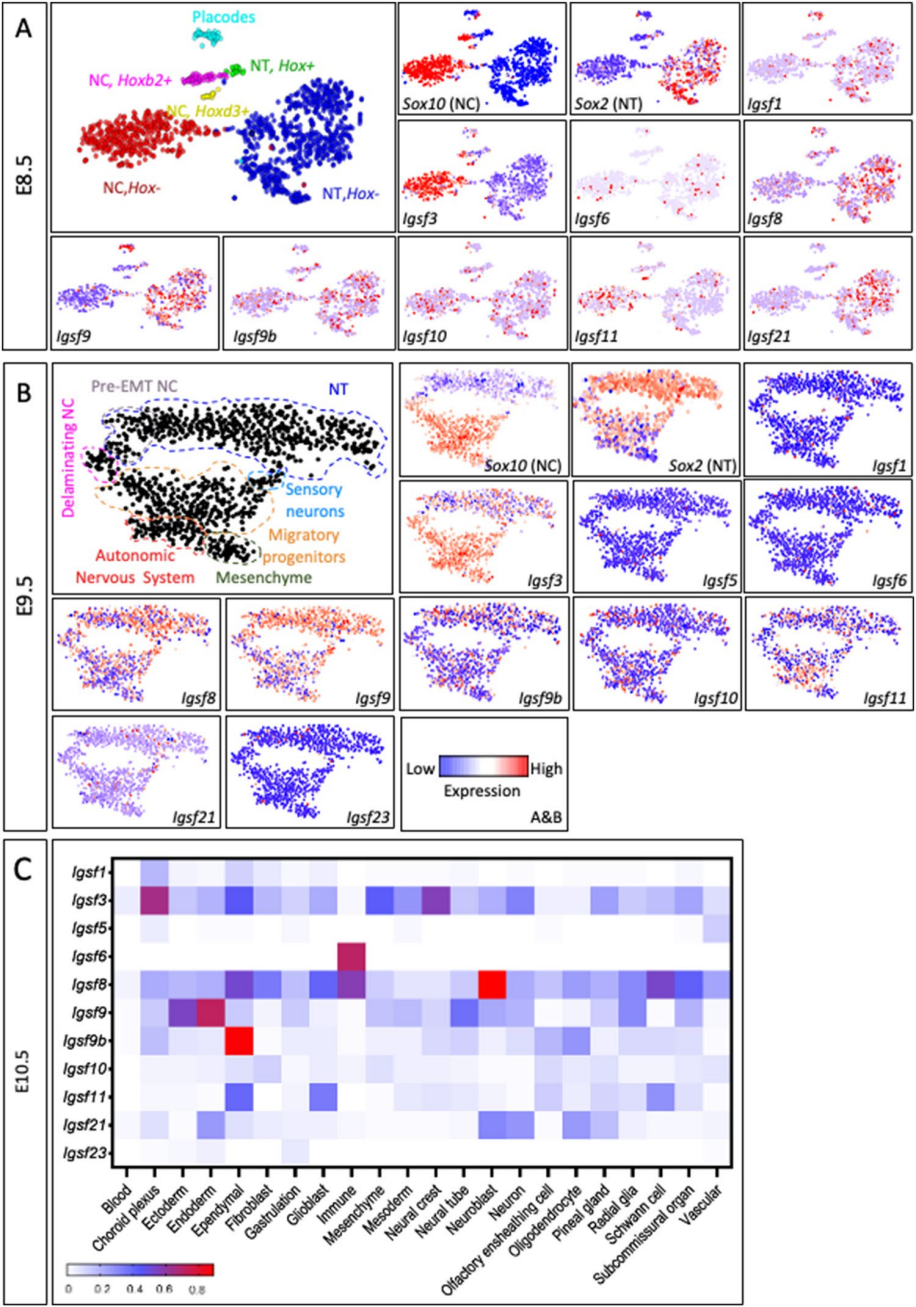
Single-cell RNAseq analysis shows that *Igsf3* is the only IGSF member that is expressed by the neural crest cells. (**A**) Large panel in the left upper corner displays the t-SNE embedding showing the cell clusters of E8.5 Wnt1-Cre; R26^Tomato^embryo. The image is adopted from the original analysis and annotations of the source data, of which our analysis is based on^23^. The early neural crest comprises of three spatially distinct clusters including a *Hox*-negative (–) corresponding to the anterior cranial neural crest, *Hoxb2*-positive ( +) corresponding to the mandibular level, and *HoxD3*-positive ( +) corresponding to the post-otic (including cardiac and vagal) streams of the neural crest^23^. Single cell RNA sequence analysis reveals the expression of different *Igsf* members in the distinct cell clusters defined respectively by *Sox10* (NC cells) and *Sox2* expression (NT cells). *Igsf3* is the only family member expressed solely by the neural crest cells at E8.5. The other IGSF members are expressed about equally by both cell clusters or preferentially by the neural tube cell cluster. Expression of *Igsf5* and *Igsf23* was not detected at E8.5. (**B**) Large panel in the left upper corner displays the t-SNE embedding showing the cell clusters of mouse E9.5 Wnt1Cre/R26R^Tomato^ embryo and reflects the spatiotemporal properties of neural crest (NC) development, as adopted from the original analysis of the source data^23^. These neural crest cell clusters are classified into the following major subpopulations such as pre-EMT, delaminating and migrating neural crest, sensory neurons, autonomic neurons, and mesenchyme^23^. Single cell RNA sequence analysis at E9.5 represented as t-SNE plot reveals the expression of various IgSF members in different neural tube (NT) and neural crest (NC) cell clusters defined by *Sox10* and *Sox2* expression, respectively. *Igsf3* is expressed in the NC and its derivative cell clusters also at this stage. *Igsf8* and *Igsf9* are predominantly expressed in the NT population*,* while *Igsf10* and *Igsf9b* are detected about equally in the NC and NT cells, and *Igsf11* shows a slight preferential expression in the NC cell cluster. (**C**) At E10.5, the choroid plexus and NC cells specifically express *Igsf3,* but other *Igsf* members are not co-expressed in these cells.

Next, we analyzed whether other IgSF members were expressed in the NC similarly to IGSF3. At E8.5, only *Igsf3* showed high exclusive expression in most of the cells in the NC cluster, while the other family members were expressed predominantly by the NT cluster (*Igsf8, Igsf9, Igsf21*) or equally in some cells in both the NC and NT clusters (*Igsf6, Igsf9b, Igsf10*). Additionally, *Igsf11* expression was found predominantly in the NC cell population but the overall percentage of the *Igsf11*-positive cells in the NC cell cluster was low (Fig. 3A). No expression of *Igsf5* and *Igsf23* was detectable at E8.5 in the used dataset. At E9.5, of the expressed *Igsf* genes, *Igsf8* and *Igsf9* were predominantly expressed in the NT population*,* while *Igsf9b* and *Igsf10* were detected about equally in the NC and NT cells, and *Igsf11* showed a slight preferential expression in the NC cell cluster (Fig. 3B) while *Igsf3* was mainly expressed by the NC cell cluster. At E10.5^25^, *Igsf3* was exclusively expressed in the choroid plexus and the NC cells, as no expression of other IgSF members was detected in these cells (Fig. 3C). Regarding the other IgSF members, high *Igsf6* expression was observed in immune cells, notable levels of *Igsf8* were detected in neuroblasts, ependymal, immune, and Schwann cells, while *Igsf9* was restricted to the ectoderm and endoderm, and *Igsf9b* expression was detected in ependymal cells (Fig. 3C). Combined, the scRNAseq data indicate that *Igsf3* is the only IGSF member that is highly expressed by the NC cells.

### Inactivation of *Igsf3* in mouse by using CRISPR/Cas9

**Figure 4:**
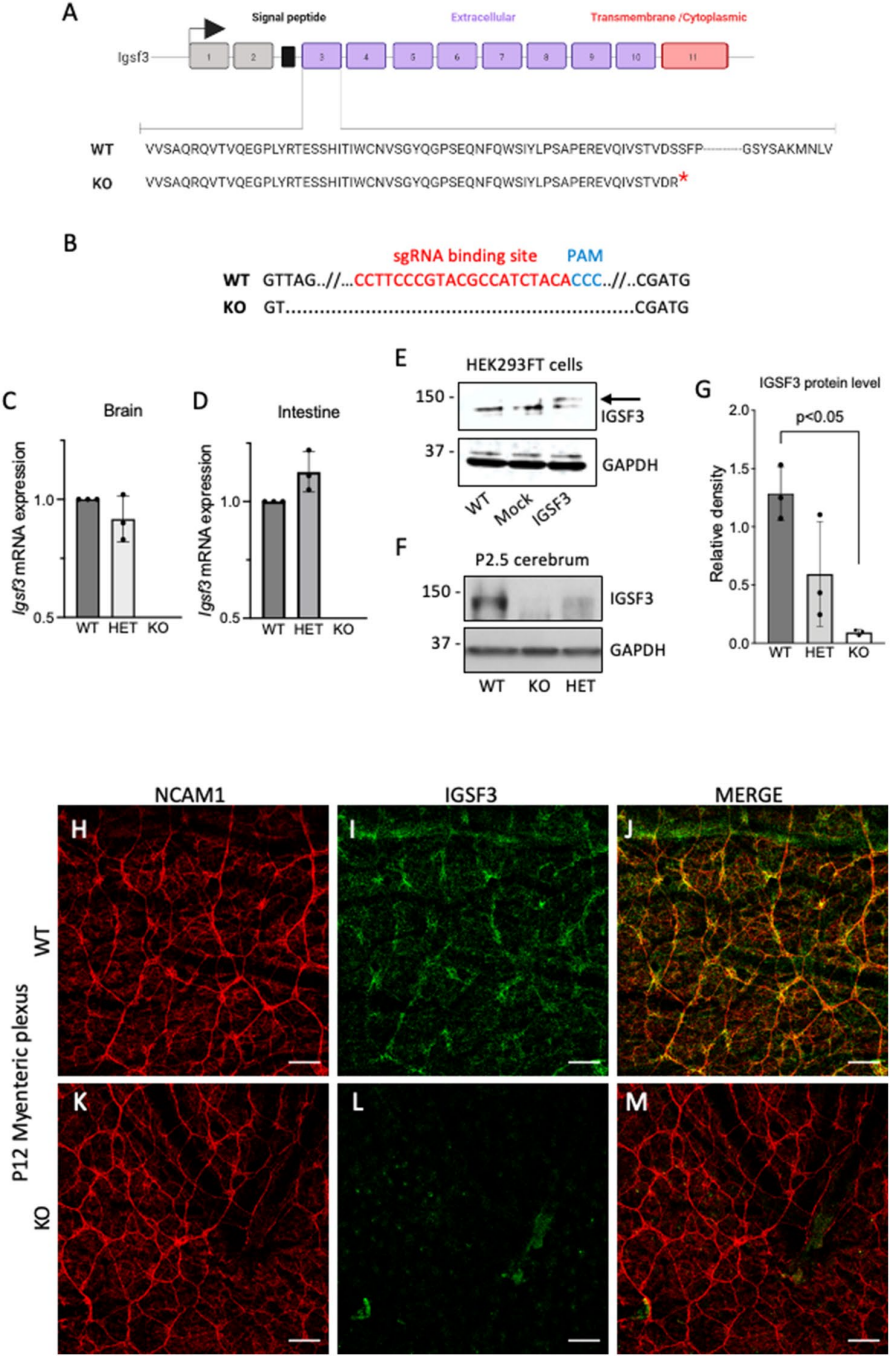
Deletion in the exon 3 *of Igsf3* gene leads near to complete loss of the IGSF3 protein. The CRISPR-Cas9 mediated genome engineering targeted exon 3 of the *Igsf3* gene. (**A**) Schematic presentation of the *Igsf3* inactivation strategy. Deleted nucleotides span across the sgRNA binding sites. The red star at the end of the nucleotide sequence indicates the pre-mature stop codon. (**B**) Gene analysis reveals a 146-nucleotide deletion in the KO allele, which resulted in a premature stop codon right after the signal peptide of *Igsf3*. *PAM*   protospacer adjacent motif. (**C**,**D**) The effect of *Igsf3* deletion at the mRNA level was evaluated by using qPCR of the P2.5 cerebrum (**C**) and intestine (**D**). In heterozygous tissues, no significant differences in the *Igsf3* levels were detected at the mRNA level whereas no *Igsf3* transcript was detected in the KO tissue samples. (**E**) HEK293FT cells were transfected with plasmid encoding the murine *Igsf3* gene. Cell extracts were prepared 48 h post-transfection and IGSF3 expression was analyzed using Western blot. WT and mock-transfected HEK293FT were used as controls. Arrow points to the band with correct molecular weight. **(F**) IGSF3 gene deletion was validated at the protein level from P2.5 cerebrum extracts by using Western blot analysis. The KO showed a nearly complete loss of protein expression while reduced expression was detected in the heterozygous brain samples. (**G**) The IGSF3 protein expression of the cerebrum samples was quantified by normalizing it to the house keeping protein GAPDH. (**H**–**M**) The successful loss of the *Igsf3* in the KO animals was further validated by immunostaining the myenteric plexus of the intestinal wall on whole mount samples from P12.5 mice using anti-NCAM1 (**H**,**K**) and anti-IGSF3 (**I**,**L**) antibodies (N = 3 for each genotype). No IGSF3 signal was detected in the KO intestines (**L**,**M**) while expression was clearly visible in the WT (**I**,**J**) and it colocalized with the NCAM1. Scale bars: (**G**–**L**)100 µm. NCAM levels in (**H**) and (**K**) have been enhanced to visualize the neuronal network since NCAM1 expression is reduced in the KO compared to the WT.

To understand the physiological role of *Igsf3,* we generated an *Igsf3* gene knockout mouse model by inactivating it using the CRISPR-Cas9 mediated genome editing system. Briefly, the in vitro transcribed small guide RNA (sgRNA) targeting the exon 3 of *Igsf3* together with *Cas9* mRNA was injected into the pronuclei of FVB/NRj zygotes to generate potential founders that were screened by PCR-based analyses followed by Sanger sequencing. Genotyping revealed a 146-nucleotide deletion in the targeted exon 3, which was confirmed by Sanger sequencing**.** The *in-silico* translation of the KO DNA sequence by using the Serial Cloner software (http://serialbasics.free.fr/Serial_Cloner.html) showed that this deletion causes a frameshift mutation resulting in a premature stop codon (Fig. 4A,B). We first analyzed the *Igsf3* mRNA levels by using qPCR in the postnatal day 2.5 (P2.5) cerebrum and intestinal samples. No *Igsf3* mRNA was detected in the KO animals while no difference was observed in the mRNA levels between the heterozygous (HET) and wild type (WT) littermates (Fig. 4C,D). To validate the loss of IGSF3 expression at protein level, we first verified that our anti-human IGSF3 antibody also recognizes the mouse IGSF3 protein. To this end, we transfected the human embryonic kidney (HEK293FT) cells that express low levels of IGSF3 with plasmid encoding the murine *Igsf3* gene. Forty-eight hours post-transfection we prepared extracts from the WT, mock-transfected, and *Igsf3*-transfected HEK293FT cells and analyzed the expression by using Western blot. Only the *Igsf3*-transfected cells showed IGSF3 protein expression (arrow in Fig. 4E, Supplementary Information) confirming that the antibody also recognizes the murine protein. Next, we performed the analysis of IGSF3 protein expression in the P2.5 cerebrum extracts of WT, heterozygous, and KO pups. Our results show nearly a complete loss of protein in KOs, whereas the heterozygous animals showed an average reduction of 46% (Fig. 4F,G, Supplementary Information). In addition, *Igsf3* loss in the KO animals was confirmed at the protein level using whole mount immunofluorescence staining of the P12 intestine. In the P12 WT animals, IGSF3 expression was detected in the myenteric plexus of the small intestine where it colocalized with the neural marker NCAM1 (Fig. 4H–J). No IGSF3 expression was detected in the KO intestinal wall (Fig. 4K–M).

### *Igsf3* KO impairs neural crest cell migration and development of intestinal muscularis propria

**Figure 5:**
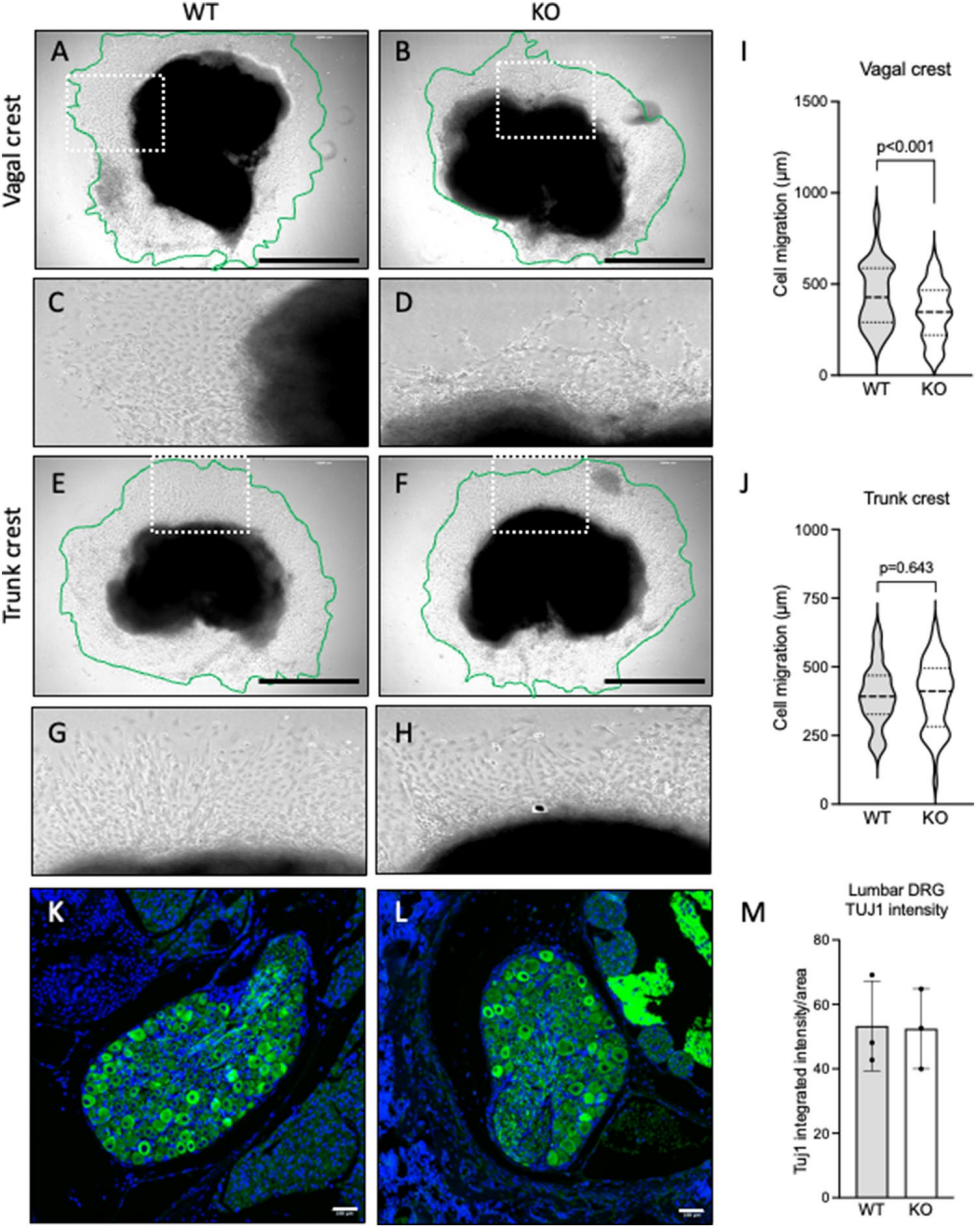
Loss of *Igsf3* impairs neural crest cell migration. (**A**–**J**) E9.0 WT and KO vagal (collected at somite level 1–7) and trunk (collected at somite level 9–12) neural tube explants were cultured on fibronectin-coated cover slips for 48 h after which they were imaged. (**A**,**B**) Vagal neural crest explants isolated from WT (**A**) and KO (**B**) embryos. (**C**,**D**) Panels show the zoomed-in view of the boxed areas in (**A**) and (**B**). (**E**,**F**) Trunk neural crest explants isolated from WT (**E**) and KO (**F**) embryos. (**G**,**H**) Panels show the zoomed-in view to the boxed areas in (**E**) and (**F**). (**I**,**J**) Quantification of the neural crest cell migration. The area containing the migrating cells is marked with a green line, and the migration distance between the outer edges of the halo and the explant (black) was measured. The vagal neural crest cells of the KO explants migrated significantly less than the WT neural crest cells (**I**) while no difference was detected in the trunk neural crest cell migration between the WTs and KOs (**J**) (N = 4 for each genotype). (**K**,**L**) Representative images of the dorsal root ganglia in WT (**K**) and KO (**L**) pups at P12.5 (N = 3 for each genotype). Neurons are visualized by Tuj1-staining (green) and nuclei with DAPI (blue). (**M**) Quantification of Tuj1-positive neurons in the dorsal root ganglia showed no difference between the WT and KO samples. Scale bars: (**A**,**B**) and (**E**,**F**) 1000 µm, **(K**,**L**) 100 µm.

**Figure 6:**
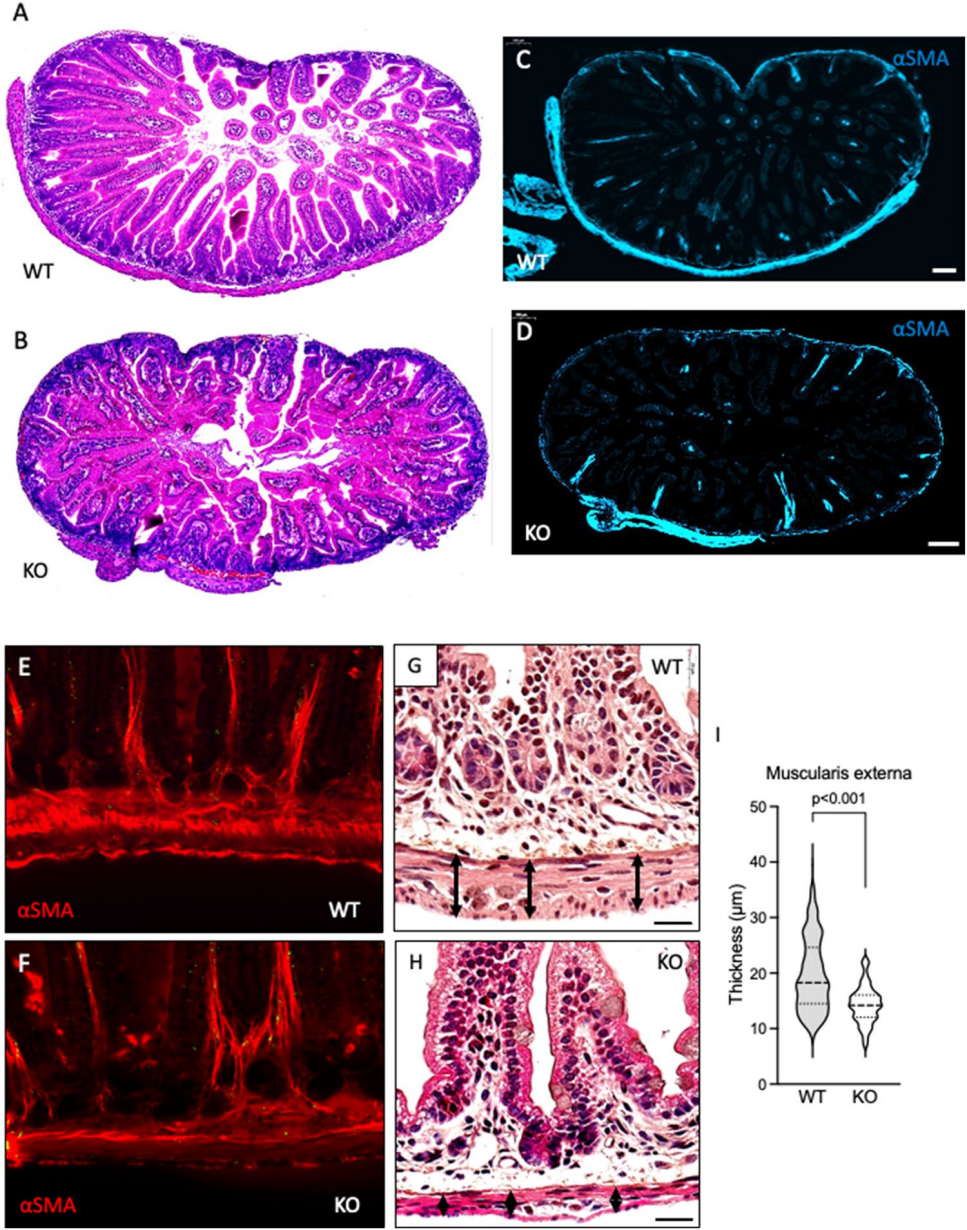
Loss of *Igsf3* results in impaired development of intestinal muscularis propria. (**A**,**B**) Small intestines collected from the P9 WT and KO pups (N = 1 for each genotype) and stained with hematoxylin and eosin (H&E). (**C**–**F**) To visualize the developing muscle layers of the small intestine the sections were stained with antibodies against αSMA (blue in (**C**,**D**), red in (**E**,**F**). (**C**,**D)** show the whole intestinal section while the panels (**E**,**F**) show the higher magnification images**.** (**G,H)** Higher magnification H&E staining from a representative sample of the small intestine from WT (**G**) and KO (**H**) P12.5 pups (N = 3 for each genotype). The arrows indicate the thickness of the muscularis externa (muscularis propria). (**I**) Quantification of the thickness of the developing enteric muscularis externa from P12.5 pups shows a significant reduction in the KO intestine as compared to samples from WT littermate controls. N = 3 for each genotype. Data was measured from two sections/intestine, four images/section, and 10 measurements points per image. Scale bars; (**C**,**D**) 100 µm, (**G**,**H**) 20 µm.

The *Igsf3* KO pups were born in Mendelian ratios and so far, have not shown deviations from the normal lifespan (data not shown). The high IGSF3 expression in NC cells in early embryonic development suggests its involvement in neural crest and peripheral nervous system development. To address this, we first performed an in vitro migration assay using the vagal and trunk neural crest explants isolated at E9.0 from the WT and KO embryos. The explants were cultured on fibronectin-coated plates and migration of NC cells was followed for 48 h after which the explants were imaged (Fig. 5A–H) and the distance of the migrated cells from the explants was quantified (Fig. 5I,J). The migration length of the KO derived vagal NC cells was significantly reduced as compared to the WT derived vagal NC cells (Fig. 5A–D,I). Interestingly, no difference was observed in the migration of the trunk NC cells between the KO and WT (Fig. 5E–J). In line with this, we immunostained the neurons of the lumbar dorsal ganglion, which are derivatives of the trunk NC cells, and no difference in the intensity of the Tuj1 expression was detected between the KO and WT (Fig. 5K–M). As the vagal neural crest contributes to the enteric neurons and glia, we focused our investigation on the effects of the loss of *Igsf3* in the small intestine. At P9 the cross sections of the KO small intestine showed disorganized structure with rudimentary outer muscle layer compared to the WT counterpart (Fig. 6A,B). The staining using anti-alpha smooth muscle actin (αSMA) antibodies confirmed the observation at P9 (Fig. 6C–F). More detailed analysis at P12.5 showed reduced thickness of the muscularis externa of the proximal small intestine in the KO (Fig. 6H) compared with the WT littermate controls (Fig. 6G). The quantification revealed a statistically significant reduction in the thickness of muscularis externa in the KO intestines (Fig. 6I).

**Figure 7:**
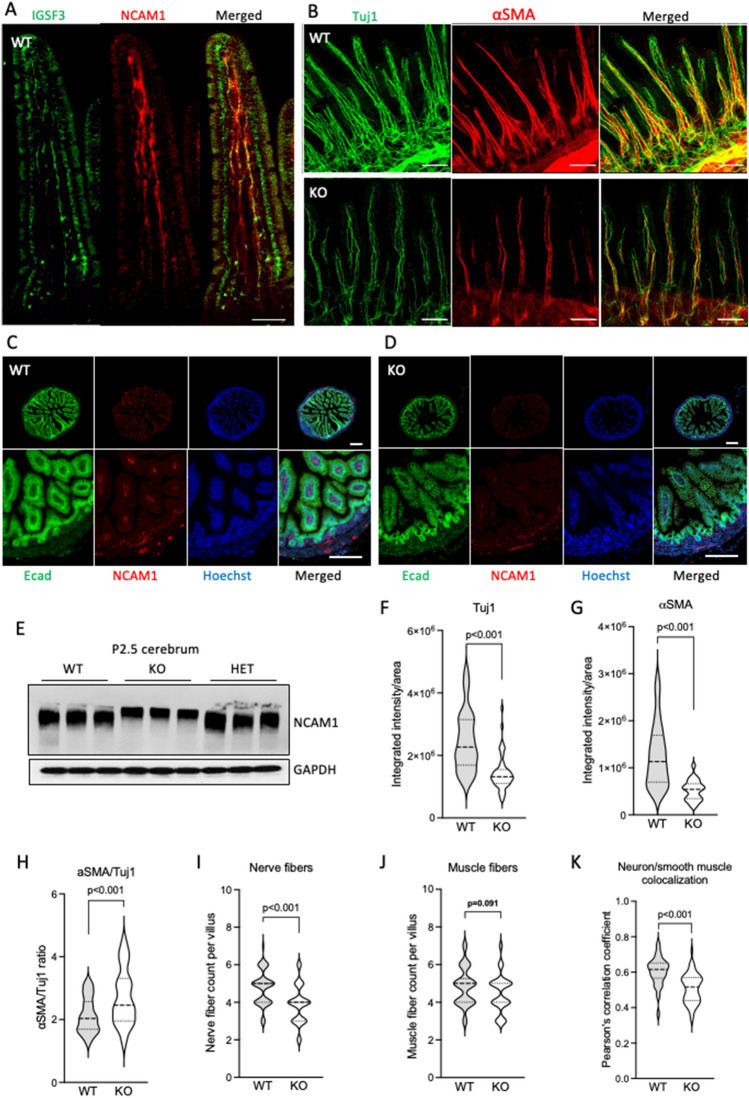
*Igsf3* KO results in downregulation of neuronal and smooth muscle markers and disorganized innervation of the small intestinal villi. (**A**) Confocal images of the WT P12.5 small intestinal villi after wholemount staining using antibodies against IGSF3 and NCAM1. IGSF3 (green) colocalizes with NCAM1 (red), a neuronal marker, in the neurons of the small intestinal villi (Merged). (**B**) Representative images of the intestinal villi from P12.5 WT (upper panels) and *Igsf3* KO (lower panels) pups stained with antibodies against the neuron marker Tuj1 (green) and smooth muscle marker αSMA (red) on whole mount samples. (**C**,**D**) Representative images of the intestine from P12.5 WT (**C**) and *Igsf3* KO (**D**) pups stained with antibodies against the E-cadherin (Ecad, green) and NCAM1 (red) on whole mount samples. Cell nuclei are visualized by Hoechst (blue). While no difference was seen in the E-cadherin expression between the WT and KO intestine, NCAM1 expression was substantially reduced in the KO compared to the WT. (**E**) Western blot analysis of P2.5 cerebrum extracts shows reduced expression of NCAM1 in KO samples also in the brain. Housekeeping protein GAPDH was used as a loading control. (**F**–**K**) Quantification from P12.5 intestinal immunostained images. (**F**) Expression of both Tuj1 and (**G**) αSMA was significantly reduced. (**H**) The αSMA:Tuj1 ratio was significantly increased in the KO villi as compared to WTs. (**I**) Counts of nerves per villus was significantly decreased in the KO villi as compared to the WT intestine, (**J**) but no difference was observed in the muscle fiber counts. (**K**) The colocalization of αSMA-Tuj1 was reduced in the KO villi as compared to WT intestines. Scale bars: (**A**–**D**) 100 µm.

Next, we investigated the detailed localization of IGSF3 in the intestinal villi by using immunostaining. Our results show that the expression of IGSF3 in the villi co-localizes with the NCAM1-positive neurons (Fig. 7A) similar to what we detected in the myenteric plexus (Fig. 4H-J). These results, together with the observed thinner muscularis externa in KO intestine, prompted us to hypothesize that IGSF3 in the villi is required for the neurons to support the proper development of the smooth muscle cells. To this end, we visualized the smooth muscle cells and neurons of the villi in the whole-mount small intestine at P12 by using immunostaining with antibodies against Tuj1 and αSMA. Our results show reduced expression of both markers in the KO villi as compared to the WT (Fig. 7B). Consequently, we utilized neuronal cell adhesion molecule 1 (NCAM1) as an additional neuronal marker to visualize neurons in the P9 intestine. The samples were co-stained with an antibody against the epithelial adhesion molecule E-cadherin (E-Cad), which marks the lining of the small intestinal villi. While the transverse sections of the WT villi readily show NCAM-positive neurons inside each villus, they are markedly reduced within the KO intestine (Fig. 7C,D). Since IGSF3 is known to be expressed in the brain^12^ we performed Western blot analysis of P2.5 cerebrum extracts, which also showed reduced NCAM1 expression in KOs compared to heterozygous and WT animals (Fig. 7E, Supplementary Information) further supporting our results on IGSF3 impacting neuronal development. Of note, the NCAM1 expression was also reduced in the myenteric plexus of the KO mice shown in Fig. 4H and K, but the NCAM1 expression is enhanced to visualize that although defected, the neuronal network in the KO mice is not lost.

Next, we performed several additional quantifications to get a more in depth understanding of the intestinal villus phenotype. First, measurement of the integrated intensity of the fluorescent signal confirmed a significant reduction for both Tuj1 and αSMA in the KO intestines as compared to the WT (Fig. 7F–G). Moreover, quantitative measurement of the ratio between the smooth muscle fibers (αSMA) and the neurons (Tuj1) in the villi revealed an increased ratio of αSMA positive staining over Tuj1 in the KO villi (Fig. 7H). This suggests that the knockout villi contained either fewer neurons per smooth muscle fiber as what is present in the WT villi, or alternatively, that the Tuj1-levels per neuron are more reduced than the respective reduction in the αSMA expression measured in the smooth muscle cells. To understand this, we quantified the numbers of nerve and muscle fibers in the villi and observed a significantly reduced number of nerve fibers per KO villi as compared to the WT villi (Fig. 7I). No difference in the muscle fiber counts per villi was observed (Fig. 7J). Lastly, we measured whether the alignment of the neurons with the smooth muscle was altered by quantifying co-localization of the two markers. Indeed, the results show impaired alignment in the KO intestine as compared to the WT (Fig. 7K). In sum, while the expression of aSMA in the muscle fibers was reduced (Fig. 7G), the number of the fibers was not changed (Fig. 7J) indicating that the individual fibers expressed lower levels of SMA. On the other hand, we find that while the expression of Tuj1 was reduced (Fig. 7F), the amount of the nerve fibers was also reduced (Fig. 7I). These results suggest that IGSF3 is required for the proper development and organization of the innervation process of the intestinal villi.

## Discussion

Several cell adhesion molecules of the immunoglobulin superfamily (IgSF), including the NCAM^26^ and members of the L1 family of neuronal cell adhesion molecules^27^, play crucial roles in the development of the nervous system. However, despite extensive study of selected IgSF proteins, the IgS family remains poorly characterized^28^. Here, by using immunohistochemistry and scRNAseq analyses we show that, in addition to the expression of the immunoglobulin super family member 3 (IGSF3) in the central nervous system, as reported by a previous study^12^, it is strongly expressed in the early, premigratory, and migratory NC cells, and subsequently in a broad selection of tissues that fully or partially originate from the NC such as teeth, the craniofacial skeleton, peripheral ganglia as well as the enteric nervous system^3^.

The neural crest is a transient embryonic cell population with extraordinary migratory abilities, enabling NC cells to migrate across the embryo and colonize different tissues^6^. The NC cells of the vagal axial level give rise to the enteric nervous system as they enter the developing gut from the anterior end, and as it extends, continue to further populate posterior parts of the intestine^29^. To understand the role of *Igsf3* in migrating neural crest and its derivatives, we generated an *Igsf3* KO mouse model using the CRISPR-Cas9 genome editing system^23^ that resulted in a 146-nucleotide deletion in exon 3, which led to a complete loss of protein expression. Collectively, our phenotypic analysis showed reduced migration of the vagal NC cells and impaired development of its derivative, the enteric nervous system.

Immunostaining confirmed that it is the enteric neurons that express IGSF3 during normal embryonic and postnatal development. Analysis of the proximal small intestine at P12 revealed defects in formation of the muscularis externa and the ability of smooth muscle cells to align with their intestinal neuronal counterparts, indicating an overall disorganized innervation of the enteric villi due to loss of *Igsf3*. The intestinal neurons and smooth muscle are intimately connected and the ENS is the key-regulator of intestinal motility^30^. The age-related loss of enteric neurons associates with the increased incidence of intestinal motility problems including delays in gastric emptying and longer intestinal transit time with associated fecal stasis^31^. Diverticular disease (DD) is an intestinal neuropathy, pathogenesis of which involves intestinal motor disturbances and an underlying enteric neuromuscular pathology. Patients with DD show significantly reduced neuronal density, decreased intramuscular nerve fibers, and abnormal intestinal motility pattern^32, 33^. As the changes in ENS affect intestinal muscles and vice versa, we suggest that the effects we detected in the *Igsf3* KO animals are due to the loss of function of IGSF3 in enteric neurons. Consequently, we hypothesize that the reduced thickness of the muscularis externa is a secondary effect due to the defects in the myenteric plexus and disorganized neurons in the intestinal villi. However, revealing the molecular mechanism and distinguishing between these functions will require further experimentation outside the focus of this work.

We also observed, using an in vitro preparation of neural tube explants, impaired migration of the vagal NC cells. This may be caused by a defect in the ability to form sufficient amounts of NC cells, a delay in emigration, or an impaired migration function as epithelial-to-mesenchymal transition and migration are both enabled by turnover of cell adhesion molecules, which facilitates rapid cellular rearrangements^34–36^. As cells migrate, adhesion to the substrate induces cytoskeletal remodeling to drive membrane protrusion and cell spreading. The actin cytoskeleton and cell-to-cell contacts in collective migration allow cells to couple mechanically and chemically^37^. Consequently, this earlier phenotype of impaired NC cell migration may result in decreased numbers of NC cells reaching the gut to form the enteric nervous system, and defects in co-localization may well be caused by a combination of this and the impaired IGSF3-mediated adhesion of the neurons to the smooth muscle fibers in the KO villi leading to disorganized neuronal network. Our results are in line with previous reports showing that the loss of function of IgSF cell adhesion proteins (CAMs) lead to defective neuronal migration and affects axonal growth^38^. Moreover, IGSF3 was found to regulate axonal growth and branching and accumulate in the axon terminals^12^. In addition, IGSF3 was recently reported to drive glioma progression via synaptic remodeling and brain network hyperactivity^39^. For future studies, using our newly generated *Igsf3* knockout mouse model in combination with additional molecular approaches, it will be interesting to investigate the mechanistic details of how IGSF3 regulates adhesion in the neural crest and the enteric nervous system neurons.

Aside from controlling bowel motility and epithelial secretion, the ENS also interacts with enteroendocrine cells to modulate the intestinal immune response, and transmission of extrinsic nerve impulses, epithelial proliferation, and repair. While neurons of the ENS are the primary controllers of gastrointestinal functions, intercellular communication with enteric glia and non-ENS cell types such as macrophages and the smooth muscle cells is an essential aspect of their function^40, 41^*.* Gut motility, including peristalsis waves that move the digested material through the gut, and segmentation movement, which mixes food with digestive enzymes and bile, is regulated through crosstalk with the smooth muscle cells. Human bowel motility disorders include life threatening conditions like Hirschsprung disease and chronic intestinal pseudo-obstruction (CIPO) syndrome^41^, highlighting the clinical relevance of research on neuro-muscular interactions within ENS development. Taken together, we demonstrate that the developing tissues of the neural crest express IGSF3, which is essential for vagal neural crest migration and formation of proper connections between intestinal neurons and smooth muscle cells during intestinal development.

## Methods

### Immunohistochemistry

The tissue sections were processed using the TSA kit (Perkin Elmer) according to the manufacturer’s instructions. The deparaffinization of fixed tissues were performed by immersing the slides in tissue clear and rehydrated with graded ethanol (100–70%) and water. The heat-induced antigen retrieval was performed using citrate buffer (1.8 mM citric acid, 8.2mM sodium citrate, pH 5.0). The slides were treated with 3% H_2_O_2_-MetOH for 10 min to inactivate endogenous peroxidase activity. The tissues were blocked for 30 min with TNB buffer (0.1M Tris–HCL (pH 7.5), 0.15M NaCl, 0.5% blocking reagent from TSA kit). The primary antibodies were incubated overnight at 4 °C and biotinylated secondary antibody for 60 min at room temperature. The primary antibodies include polyclonal rabbit IGSF3 (1:750 HPA036305, Sigma-Aldrich), polyclonal mouse SOX10 (1:500 SC-365692, Santa Cruz). The reddish brown color was the stain generated by secondary biotinylated antibodies which includes goat-anti rabbit IgG (E0432, Dako) and goat-anti mouse IgG (E0433, Dako). The washes were performed with TNT (Tris/NaCl/Tween 20) buffer. The Mayer’s hematoxylin (S3309, Dako) was used to counterstain the tissue sections. For tissue morphological assessment, hematoxylin and eosin staining was performed. Whole stained slides were digitally scanned using a slide scanner (3Dhistec).

### Generation of the *Igsf3* KO mouse model

The FVB/NRj mice were obtained from the Janvier labs (France) and used in the study for genome editing. We confirm that all experiments were performed in accordance with relevant guidelines and regulations. The Committee for animal experiments of the District of Southern Finland approved the animal experiments under the licenses ESAVI/403/2019 (valid 1.4.2019–31.3.2022), ESAVI/11614/2022 (valid 1.4.2022–30.8.2022), and ESAVI/10262/2022 (valid 4.5.2022–4.5.2025). The reporting in the manuscript follows the recommendations in the ARRIVE guidelines (https://arriveguidelines.org/arrive-guidelines). We confirm that this study does not involve human participants, personal data, human cells, or tissues.

*Igsf3* inactivation in mouse was performed according to the published in vivo CRISPR/Cas9 methods^42^. The *Igsf3* gRNA (25 ng/µl, 5’-GTGTAGATGGCGTACGGGAAGG-3’), targeting the exon 3 (Exon ID-ENSMUSE00000456959) immediately after the signal peptide was obtained from mouse genome-wide arrayed lentiviral CRISPR/Cas9 gRNA libraries (Sigma-Aldrich/Merck). The gRNAs were in vitro transcribed and dissolved in Tris–EDTA buffer.The Cas9 mRNA (25 ng/µl Sigma Aldrich) and gRNA were co-injected into the pronuclei of FVB/Nj (Janvier Labs, France) zygotes. Transplantation of injected zygotes to oviduct of pseudopregnant recipient females gave rise to two founders. Sanger sequencing was performed on all the founders (F0) and F1 offspring of two founders that were used to generate *Igsf3* knockout mice. The selected founder lines had a 146- and 180- nucleotide deletion in the *Igsf3* gene that causes a frameshift resulting in a premature stop codon right after the signal peptide (Serial Cloner software).

### Genotyping

The genomic DNA from ear punctures was isolated using the NucleoSpin® Tissue according to manufacturer’s protocol. The genotyping primers were designed to span 128 nucleotides upstream and 264 nucleotides downstream of the targeted *Igsf3* exon 3. The targeted region was amplified by PCR using the Phusion DNA polymerase using the following primers (5′-CTTGGAAAGGGCCAGTAACC-3′ and 5′-CCTGGATCTGGCCTTGATAA-3′) resulting in 770 nucleotide long products. The cycle conditions were 98 °C 1 min, followed by 98 °C 10 s, 57.6 °C 30 s, 72 °C 30 s (30 cycles), then 72 °C for 5 min and stored at 4 °C. Sanger sequencing of PCR amplicons was performed to identify the editing events such as insertion, deletion, and frameshift mutation. The sequence analyses were performed using the SnapGene Viewer and Serial Cloner. The genotype of the animals was analyzed using Sanger sequencing until the F3 generation. After F3 generation, all the samples were analyzed by gel electrophoresis.

### Plasmid construction

The plasmids were generated using the Genome Biology Unit core facility cloning service (ORFeome Library; Genome Biology Unit supported by HiLIFE and the Faculty of Medicine, University of Helsinki and Biocenter Finland, https://www.helsinki.fi/en/researchgroups/genome-biology-unit/orf-libraries). The mouse *Igsf3* ORF (CAT#: MR211786, Origene) sequence was amplified using the Gateway-tailed primers. Briefly, the gateway-tailed entry clone was transferred into the pLenti7.3/V5-DEST (ampicillin) using the standard LR reaction (gateway cloning) protocol.

(Gateway-tailed **Forward Primer:** 5′ GGGGACAACTTTGTACAAAAAAGTTGGCACCATGAAG TGCTTTTTCCCAGTGTTGAGCT, Gateway-tailed **Reverse Primer:** 5′ GGGGACAACTTTGTACAAGAAAGTTGGCAAGTCAATGGC TCCCGGGTGGATGCT).

### Expression of murine IGSF3 in human HEK293FT cells

Human embryonic kidney (HEK293FT) cells (ATCC) were cultured in DMEM high glucose (4.5 g/L) with L-glutamine (Gibco) supplemented with 10% FBS (Gibco), 100 U/mL penicillin, and 100 μg/mL streptomycin (both from BioNordika). HEK293FT cells were plated one day before transfection and were approximately 60% confluent on the day of transfection. The murine *Igsf3* plasmid (ORFeome Library) was transfected into HEK293FT cells by using the FuGENE6 transfection reagent (Promega) at a 3:1 ratio of FuGENE6 transfection reagent to DNA according to the manufacturer’s protocol. Cells were incubated for 48 h, lysed, and the extracts were analyzed for IGSF3 expression by Western blotting. Mock-transfected cells (plasmid without *Igsf3)* and WT HEK293FT cells were used as controls.

### Immunoblotting

The tissue samples were snap frozen using liquid nitrogen and stored at – 80 °C. The frozen samples were transferred for homogenization to tubes containing zirconium beads (MB2Z015, Bioline) with RIPA buffer (50mM Tris HCl pH 7.4, 150mM NaCl (Sigma-Aldrich), 1% Sodium Deoxycholate (Sigma-Aldrich), 1% SDS (Sigma-Aldrich), 2% Octyl-β-D-Glucopyranoside (Sigma-Aldrich) with protease and phosphatase inhibitors (both from Roche) and homogenized using the following program: 2*3500 rpm for 15 s with 10 s’ delay. The homogenized samples were incubated in ice for 20 min and centrifuged at 4 °C for 20 min at 13,000 rpm. The Pierce™ BCA Protein Assay Kit (Thermo Fisher Scientific) was utilized for determining the protein concentration. The supernatant was collected and diluted in the SDS Laemmli loading buffer (supplemented with 5% β-mercaptoethanol), boiled for 5 min at 95 °C and electrophoresed using NuPAGE Bis–Tris Gels (Invitrogen, Thermo Fisher Scientific), and then transferred onto PVDF membranes (Bio-Rad) using the Transblot Turbo transfer system (Bio-Rad). The blocking was performed for 60 min at RT using non-fat dry milk (5%) in TBS-T buffer. The blocking buffer was used as a diluent for the primary anti-IGSF3 (AF4788, R&D Systems, 1:1000), anti-GAPDH (14C10, Cell Signaling Technology, 1:50,000) and the secondary antibodies (goat anti-rabbit HRP, Dako, 1:1000) and blotted at 4 °C for 16 h and 1 h at room temperature.

### Quantitative RT-PCR

The TriSure reagent (Bioline) was used to isolate the total RNA from frozen tissue samples. The samples were transferred for lysis to tubes containing zirconium beads (MB2Z015, Bioline), homogenized using the following program: 2*3500 rpm for 15 s with 10 s delay, and incubated on ice for 2 min. After addition of chloroform the samples were centrifuged at 12000*g* for 10 min at 4 °C. The clear top layer was collected and transferred to new tubes and an equal volume of 70% EtOH was added. The samples were then processed, and the total RNA isolated using the NucleoSpin® RNA (Macherey–Nagel) according to manufacturer’s protocol. The iScript cDNA synthesis kit (Bio-Rad) was used for the reverse transcription into cDNA. Quantitative real-time PCR was performed using the Bio-Rad Laboratories CFX96 Touch™ cycler and KAPA SYBR FAST qPCR master mix (Kapa Biosystems). The gene expression was normalized to glyceraldehyde 3-phosphate dehydrogenase (*GAPDH*) housekeeping gene using the comparative Ct method. The primer sequences were *Igsf3* forward (5′-GCGGTGGGAAGATCTACGTG-3′), *Igsf3* reverse (5′-GGCTTGCAGGTCTGTGATGT-3′), *Gapdh* forward- (5′-GAGAGTGTTTCCTCGTCCC-3′), and *Gapdh* reverse (5′-ACTTTGCCACTGCAAATGG-3′).

### Neural crest migration assay

The embryos were isolated on the embryonic day 9.0 (E9.0). The vagal neural tubes (somites 1–7), and the trunk neural tubes (somites 9–12), were dissected out by removing the surrounding tissues by using micro-scissors. The neural tube explants were then placed on fibronectin coated cover slips in N2 supplemented DMEM media (both from Gibco, Thermo Fisher Scientific). The explants were incubated at 37 °C with 5% CO_2_ for 48 h during which the NC cells migrated out of the NT explant and formed a halo. The migration length was measured as the furthest distance the NC cells had reached away from the explant at 48 h. The measurements of each halo consisted of twenty-five data points evenly covering the entire halo of NC cells (Fig. 5A–D). The images were acquired at 48h using the EVOS™ FL digital inverted fluorescence microscope (Invitrogen). The images were analyzed by using the ImageJ software (http://rsb.info.nih.gov/ij/, 1997–2009; Rasband, W.S., ImageJ, U. S. National Institutes of Health, Bethesda, MD, USA).

### Tissue collection and processing

The post-natal day 12 (P12) pups were anesthetized using ketamine/xylazine combination and perfused with 1% paraformaldehyde (PFA) in PBS by cardiac puncture. The intestines were excised and fixed overnight with 4% PFA at 4 °C. The tissues were washed with PBS and processed for paraffin embedding using the manufacturer’s protocol.

### Immunofluorescence staining

The post-natal day P9 (P9) pups were anesthetized using ketamine/xylazine combination and perfused with 1% paraformaldehyde (PFA) in PBS by cardiac puncture. The intestines were excised and fixed overnight with 4% PFA at 4 °C. The tissues were processed using an automated tissue processor, paraffin embedded, cut into 5-μm-thick sections, and allowed to dry overnight. Immunofluorescent staining of paraffin sections was performed following standard protocol with xylene-alcohol deparaffination series followed by heat-induced antigen retrieval in 20 mM Tris–HCl, 1 mM EDTA buffer at pH 8.5. After cooling to room temperature, tissue sections were blocked 1 h in 50 mM Tris–HCl, 100 mM NaCl, 0.1% Tween-20, at pH 7.5 (TNT) supplemented with 10% FBS, followed by incubation with primary antibodies at 4°C overnight, TNT washing, and 1 h incubation with secondary antibodies. Primary antibodies were used as follows: E-Cad (BD61018, 1:500, BD Biosciences), NCAM1 (ab5032, 1:1000, Merck). Detection was performed by using Alexa Fluor-conjugated secondary antibodies (1:400; Jackson Immuno Research Laboratories). Additionally, fluorescent dye Hoechst (Invitrogen) was used at 1:1000 dilution to visualize the cell nuclei. Epifluorescence imaging was performed with Zeiss Axio Imager.M2 outfitted with Hamamatsu Orca Flash 4.0 LT B&W camera and Zeiss Zen 2 software.

### Hematoxylin and eosin staining

The paraffin sections were deparaffinized with xylene followed by rehydration in ethanol series. The samples were stained with Mayer’s Hematoxylin (Histolab, Sweden), rinsed, and further stained with Eosin (Histolab, Sweden). Finally, the hematoxylin–eosin-stained samples were dehydrated, and mounted with Pertex medium.

### Whole-mount immunostaining

The post-natal day 12 (P12) pups were anesthetized using ketamine (50 mg/mL)/medetomidine (1 mg/mL), trans-cardially perfused with 1% PFA/PBS, followed by the quick dissection of the small intestine in ice-cold PBS. The intestine was cut longitudinally under the stereomicroscope before being pinned onto the silicon plates and fixed with 4% PFA/PBS overnight at 4 °C. Small rectangular piece of intestine (0.5 × 1 cm) was cut out for the whole-mount staining with all the incubations and washes performed in 12-well plates on the shaker platform (30 rpm). The tissues were washed with PBS and treated with the blocking buffer (5% fetal bovine serum, 0.3% Triton-X-100 in PBS) for 1 h at room temperature. The primary antibodies against NCAM1 (ab5032, 1:500, Merck), IGSF3 (AF4788, 1:750, R&D Systems),Tuj1 (AB18207, 1:1000, Abcam) and αSMA-CY3^43^ (C6198, 1:300, Merck) were applied for 48 h at 4 °C followed by washes with 0.3% Triton-X-100 in PBS for the whole day and the secondary antibody donkey anti-rabbit Alexa Fluor 647 (A32795, 1:400, Thermo Fisher Scientific) or donkey anti-goat Alexa Fluor 488 (705–546-147, 1:400, Jackson ImmunoResearch) overnight at 4°C followed by washes for 3–4 h using 0.3% Triton-X-100 in PBS. After the secondary antibody incubation and washes, the tissues were post-fixed with 1% PFA for 10 min at RT followed by PBS wash. The rectangular piece of intestine was pinned to the silicon plate and cut into thin strips under the stereomicroscope, which were then mounted with Shandon Immu-Mount (Thermo-Scientific) and covered with coverslip. Confocal images were taken with the Leica SP8X, Zeiss LSM780 and 880 equipped with appropriate lasers and LAS X or Zeiss Zen Black software.

### Image analysis

The thickness of the P12.5 intestinal muscle layer was measured from paraffin embedded sections of 4 µm thickness stained with Hematoxylin and eosin. To quantify the thickness, two subsequent sections per intestine, four images per section and 10 measurements points per image was analyzed (N = 3 for each genotype). Immunolabeled P12.5 intestinal whole-mounts were imaged in a 512 × 512 format using Leica SP8 X with white light laser and using a 20 × objective. For analyses, 20 random intestinal villi per mouse (3 mice per genotype) were chosen from maximum projections (z-stack: 0.2 µm/step) and analyzed using Fiji. To quantify the TUJ1 and αSMA intensities, regions of interest (ROI) were determined in Fiji through its area of selection tools by using the antibody background as a boundary of a single villus and integrated intensity measurement in the ROIs selection. To quantify the nerve/muscle fibers, the brightness of the images was adjusted in Fiji and the fibers were manually quantified at the half-height of the ROI. To analyze the colocalization of TUJ1 positive nerve fibers and αSMA positive muscle fibers, the JACoP plugin was employed to calculate the Pearson's coefficient per each ROI. The violin box plots show the distribution of individual analyzed values in the intestinal villi. Statistical significance was calculated by Mann Whitney test in GraphPad Prism 9, with p ≤ 0.05 considered as statistically significant.

### Statistics

All statistics were computed using the GraphPad Prism 8. Statistical significance between the sample groups was determined using an unpaired 2-tailed *t* test or the nonparametric Mann–Whitney *U* test. For multiple comparisons, ordinary 1-way ANOVA with Tukey’s multiple comparisons tests were used. The* P* < 0.05 value was considered statistically significant, and the data presented as mean ± SD.

### Supplementary Information

Supplementary Information.

## Data Availability

Data supporting the findings of this manuscript are available from the corresponding authors upon reasonable request. The RNAseq data was extracted from the following sites: E8.5 and E9.5 http://pklab.med.harvard.edu/ruslan/neural.crest.html^23^ and E10.5 https://www.ncbi.nlm.nih.gov/sra under accession PRJNA637987^25^.

## References

[1] Copp AJ, Greene ND, Murdoch JN (2003). The genetic basis of mammalian neurulation. Nat. Rev. Genet..

[2] Theveneau E, Mayor R (2012). Neural crest migration: Interplay between chemorepellents, chemoattractants, contact inhibition, epithelial-mesenchymal transition, and collective cell migration. Wiley Interdiscip. Rev. Dev. Biol..

[3] Perera SN, Kerosuo L (2021). On the road again: Establishment and maintenance of stemness in the neural crest from embryo to adulthood. Stem Cells.

[4] Etchevers HC, Amiel J, Lyonnet S (2006). Molecular bases of human neurocristopathies. Adv. Exp. Med. Biol..

[5] Shellard A, Mayor R (2016). Chemotaxis during neural crest migration. Semin. Cell Dev. Biol..

[6] Szabo A, Mayor R (2018). Mechanisms of neural crest migration. Annu. Rev. Genet..

[7] Barriga EH, Mayor R (2015). Embryonic cell-cell adhesion: A key player in collective neural crest migration. Curr. Top. Dev. Biol..

[8] Friedl P, Mayor R (2017). Tuning collective cell migration by cell-cell junction regulation. Cold Spring Harb. Perspect. Biol..

[9] Cavallaro U, Christofori G (2004). Cell adhesion and signalling by cadherins and Ig-CAMs in cancer. Nat. Rev. Cancer.

[10] Leshchyns'ka I, Sytnyk V (2016). Reciprocal interactions between cell adhesion molecules of the immunoglobulin superfamily and the cytoskeleton in neurons. Front. Cell Dev. Biol..

[11] Saupe S, Roizes G, Peter M, Boyle S, Gardiner K, De Sario A (1998). Molecular cloning of a human cDNA IGSF3 encoding an immunoglobulin-like membrane protein: Expression and mapping to chromosome band 1p13. Genomics.

[12] Usardi A, Iyer K, Sigoillot SM, Dusonchet A, Selimi F (2017). The immunoglobulin-like superfamily member IGSF3 is a developmentally regulated protein that controls neuronal morphogenesis. Dev. Neurobiol..

[13] Furness JB, Rivera LR, Cho HJ, Bravo DM, Callaghan B (2013). The gut as a sensory organ. Nat. Rev. Gastroenterol. Hepatol..

[14] Rao M, Gershon MD (2016). The bowel and beyond: The enteric nervous system in neurological disorders. Nat. Rev. Gastroenterol. Hepatol..

[15] Furness JB (2006). The organisation of the autonomic nervous system: Peripheral connections. Auton. Neurosci..

[16] Collier CA, Mendiondo C, Raghavan S (2022). Tissue engineering of the gastrointestinal tract: The historic path to translation. J. Biol. Eng..

[17] Furness JB (2008). The enteric nervous system: Normal functions and enteric neuropathies. Neurogastroenterol. Motil..

[18] Mercado-Perez A, Beyder A (2022). Gut feelings: Mechanosensing in the gastrointestinal tract. Nat. Rev. Gastroenterol. Hepatol..

[19] Tullie L, Jones BC, De Coppi P, Li VSW (2022). Building gut from scratch - progress and update of intestinal tissue engineering. Nat. Rev. Gastroenterol. Hepatol..

[20] Mowat AM, Agace WW (2014). Regional specialization within the intestinal immune system. Nat. Rev. Immunol..

[21] Brasseur JG, Nicosia MA, Pal A, Miller LS (2007). Function of longitudinal vs circular muscle fibers in esophageal peristalsis, deduced with mathematical modeling. World J. Gastroenterol..

[22] Furness JB (2012). The enteric nervous system and neurogastroenterology. Nat. Rev. Gastroenterol. Hepatol..

[23] Soldatov R, Kaucka M, Kastriti ME, Petersen J, Chontorotzea T, Englmaier L (2019). Spatiotemporal structure of cell fate decisions in murine neural crest. Science.

[24] Kerosuo L, Neppala P, Hsin J, Mohlin S, Vieceli FM, Torok Z (2018). Enhanced expression of MycN/CIP2A drives neural crest toward a neural stem cell-like fate: Implications for priming of neuroblastoma. Proc. Natl. Acad. Sci. U.S.A..

[25] La Manno G, Siletti K, Furlan A, Gyllborg D, Vinsland E, Mossi Albiach A (2021). Molecular architecture of the developing mouse brain. Nature.

[26] Akitaya T, Bronner-Fraser M (1992). Expression of cell adhesion molecules during initiation and cessation of neural crest cell migration. Dev. Dyn..

[27] Anderson RB, Turner KN, Nikonenko AG, Hemperly J, Schachner M, Young HM (2006). The cell adhesion molecule l1 is required for chain migration of neural crest cells in the developing mouse gut. Gastroenterology.

[28] Verschueren E, Husain B, Yuen K, Sun Y, Paduchuri S, Senbabaoglu Y (2020). The immunoglobulin superfamily receptome defines cancer-relevant networks associated with clinical outcome. Cell.

[29] Hutchins EJ, Kunttas E, Piacentino ML, Howard AGAT, Bronner ME, Uribe RA (2018). Migration and diversification of the vagal neural crest. Dev. Biol..

[30] Di Nardo G, Blandizzi C, Volta U, Colucci R, Stanghellini V, Barbara G (2008). Review article: Molecular, pathological and therapeutic features of human enteric neuropathies. Aliment. Pharmacol. Ther..

[31] Phillips RJ, Powley TL (2007). Innervation of the gastrointestinal tract: Patterns of aging. Auton. Neurosci..

[32] Hellwig I, Bottner M, Barrenschee M, Harde J, Egberts JH, Becker T (2014). Alterations of the enteric smooth musculature in diverticular disease. J. Gastroenterol..

[33] Wedel T, Busing V, Heinrichs G, Nohroudi K, Bruch HP, Roblick UJ (2010). Diverticular disease is associated with an enteric neuropathy as revealed by morphometric analysis. Neurogastroenterol. Motil..

[34] Carmona-Fontaine C, Matthews HK, Kuriyama S, Moreno M, Dunn GA, Parsons M (2008). Contact inhibition of locomotion in vivo controls neural crest directional migration. Nature.

[35] Theveneau E, Linker C (2017). Leaders in collective migration: Are front cells really endowed with a particular set of skills?. F1000Research.

[36] Leathers TA, Rogers CD (2022). Time to go: Neural crest cell epithelial-to-mesenchymal transition. Development.

[37] De Pascalis C, Etienne-Manneville S (2017). Single and collective cell migration: The mechanics of adhesions. Mol. Biol. Cell.

[38] Cohen NR, Taylor JS, Scott LB, Guillery RW, Soriano P, Furley AJ (1998). Errors in corticospinal axon guidance in mice lacking the neural cell adhesion molecule L1. Curr. Biol..

[39] Curry RN, Aiba I, Meyer J, Lozzi B, Ko Y, McDonald MF (2023). Glioma epileptiform activity and progression are driven by IGSF3-mediated potassium dysregulation. Neuron.

[40] Carbone SE (2022). Neurons, macrophages, and glia: The role of intercellular communication in the enteric nervous system. Adv. Exp. Med. Biol..

[41] Schneider S, Wright CM, Heuckeroth RO (2019). Unexpected roles for the second brain: Enteric nervous system as master regulator of bowel function. Annu. Rev. Physiol..

[42] Fernandez A, Morin M, Munoz-Santos D, Josa S, Montero A, Rubio-Fernandez M (2020). Simple protocol for generating and genotyping genome-edited mice with CRISPR-Cas9 reagents. Curr. Protoc. Mouse Biol..

[43] Gurdziel K, Vogt KR, Walton KD, Schneider GK, Gumucio DL (2016). Transcriptome of the inner circular smooth muscle of the developing mouse intestine: Evidence for regulation of visceral smooth muscle genes by the hedgehog target gene, cJun. Dev. Dyn..

