# Supplementary material for: Immunoglobulin superfamily member 3 is required for the vagal neural crest cell migration and enteric neuronal network organization
Source: Sci Rep. 2023 Oct 11;13:17162. doi: 10.1038/s41598-023-44093-8 (PMC10567708; doi:10.1038/s41598-023-44093-8)
Supplement: Supplementary file 1 — Supplementary Information. [file 41598_2023_44093_MOESM1_ESM.docx]

**Supplementary Information**

**Immunoglobulin superfamily member 3 is required for the vagal neural crest cell migration and enteric neuronal network organization**

Jayendrakishore Tanjore Ramanathan^1#^, Tomáš Zárybnický^2#^, Pauliina Filppu^1^, Hector Monzo^1^, Outi Monni^3^, Topi Tervonen^1,4^, Juha Klefström^1,5^, Laura Kerosuo^6*^, Satu Kuure^2,7*^ and Pirjo Laakkonen^1,8,9*^

Whole membrane image of the Western blot using anti-IGSF3 antibodies of HEK293FT cell extracts. Lane 1, WT HEK293FT cells; lane 2, mock-transfected HEK293FT cells; lane 3, HEK293FT cells expressing murine IGSF3. The boxed area represents the Figure 4E in the manuscript. Western blot was visualized by using the Azure 500 Imaging System (Azure Biosystems).

Whole membrane image of the Western blot using anti-GAPDH antibodies of HEK293FT cell extracts. Lane 1, WT HEK293FT cells; lane 2, mock-transfected HEK293FT cells; lane 3, HEK293FT cells expressing murine IGSF3. The boxed area represents the Figure 4E in the manuscript. Western blot was visualized by using the Azure 500 Imaging System (Azure Biosystems).

Whole membrane image of the Western blot using anti-IGSF3 antibodies of cerebrum extracts of the P2.5 wildtype (WT), heterozygous (HET), and *Igsf3* knockout (KO) pups. The boxed area represents the Figure 4F in the manuscript.

Whole membrane image of the Western blot using anti-GAPDH antibodies of cerebrum extracts of the P2.5 wildtype (WT), heterozygous (HET), and *Igsf3* knockout (KO) pups. The boxed area represents the Figure 4F in the manuscript.


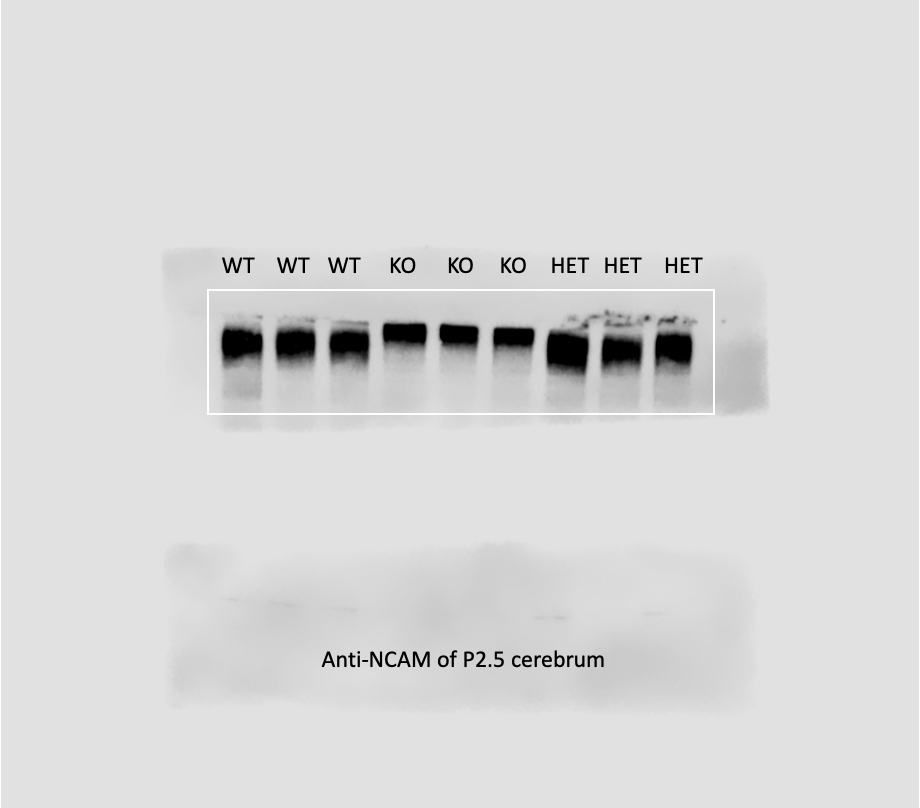


Whole membrane image of the Western blot using anti-NCAM1 antibodies of cerebrum extracts of the P2.5 wildtype (WT), heterozygous (HET), and *Igsf3* knockout (KO) pups. The boxed area represents the Figure 7E in the manuscript. Western blot was visualized by using the Odyssey imaging system (LiCOR).

Whole membrane image of the Western blot using anti-GADH antibodies of cerebrum extracts of the P2.5 wildtype (WT), heterozygous (HET), and *Igsf3* knockout (KO) pups. The boxed area represents the Figure 7E in the manuscript.
